# Conserved Transcriptional Signatures in Human and Murine Diabetic Peripheral Neuropathy

**DOI:** 10.1038/s41598-018-36098-5

**Published:** 2018-12-05

**Authors:** Brett A. McGregor, Stephanie Eid, Amy E. Rumora, Benjamin Murdock, Kai Guo, Guillermo de Anda-Jáuregui, James E. Porter, Eva L. Feldman, Junguk Hur

**Affiliations:** 1 Department of Biomedical Sciences, University of North Dakota School of Medicine and Health Sciences, Grand Forks, North Dakota, 58202 USA; 2 Department of Neurology, University of Michigan, Ann Arbor, Michigan 48109 USA

## Abstract

Diabetic peripheral neuropathy (DPN) is one of the most common complications of diabetes. In this study, we employed a systems biology approach to identify DPN-related transcriptional pathways conserved across human and various murine models. Eight microarray datasets on peripheral nerve samples from murine models of type 1 (streptozotocin-treated) and type 2 (*db*/*db* and *ob*/*ob*) diabetes of various ages and human subjects with non-progressive and progressive DPN were collected. Differentially expressed genes (DEGs) were identified between non-diabetic and diabetic samples in murine models, and non-progressive and progressive human samples using a unified analysis pipeline. A transcriptional network for each DEG set was constructed based on literature-derived gene-gene interaction information. Seven pairwise human-vs-murine comparisons using a network-comparison program resulted in shared sub-networks including 46 to 396 genes, which were further merged into a single network of 688 genes. Pathway and centrality analyses revealed highly connected genes and pathways including LXR/RXR activation, adipogenesis, glucocorticoid receptor signalling, and multiple cytokine and chemokine pathways. Our systems biology approach identified highly conserved pathways across human and murine models that are likely to play a role in DPN pathogenesis and provide new possible mechanism-based targets for DPN therapy.

## Introduction

According to the United States Centres for Disease Control and Prevention (CDC), more than 29 million Americans, over 9% of the United States population, are living with diabetes and another 86 million have prediabetes^1^. The most common microvascular complication of diabetes is diabetic peripheral neuropathy (DPN) which occurs in approximately 60% of patients and is the leading cause of non-traumatic lower-limb amputations^2,3^. DPN is characterized by distal to proximal degeneration of peripheral nerves which results in symptoms such as numbness, pain, and weakness^4^. Other than glucose control, there are no disease-modifying treatments for DPN. Understanding DPN pathology and identifying the underlying mechanisms of peripheral nerve degeneration are therefore critical to the development of new mechanism-based therapies for DPN.

Over the past decade, with the advent of high-throughput gene expression profiling assays such as microarrays and RNA-Seq, we and others have examined genome-wide gene expression changes from the peripheral nerve tissues of various diabetic murine models^5–9^ and human subjects^10,11^ with diabetes. Bioinformatics analyses of these high-throughput datasets identified numerous genes in human and murine peripheral nerves that are significantly dysregulated by diabetes. Suggested mechanisms of injury such as inflammation, oxidative stress, lipid and carbohydrate metabolism, regulation of axonogenesis, mitochondrion, and peroxisome proliferator-activated receptor (PPAR) signalling were also reported during diabetes onset and progression^6,7,9,10^.

Yet, one limitation of the previous studies, including ours, is that the analyses didn’t account for the differences in species, strains, procedure of diabetes induction, and diabetes duration. Another critical issue that hasn’t been addressed thus far is the identification of common injurious pathways and networks conserved across various mouse models of diabetes as well as between mouse and human. In this study, we reanalysed previously published DPN-related microarray datasets from human and multiple murine models using a unified analysis pipeline. Compared with the existing literature, this study provides a unique opportunity to uncover a possible common mechanism of injury shared across DPN stages, types of diabetes, and species. Such mechanisms could unravel new important therapeutic targets to treat DPN.

## Research Design and Methods

### Microarray data

All datasets used were gathered from the Diabetic Neuropathy Microarray Knowledge-Base (DNMKB; http://hurlab.med.und.edu/DNMKB/). The data from the type 1 diabetes mellitus (T1DM) model were originally generated from male DBA/2J mice treated with streptozotocin (STZ) at 10 weeks that were terminated at 34 weeks^9^. The two type 2 diabetes mellitus (T2DM) models included BKS.Cg-Lepr^db/db^ (*db*/*db*) mice and BTBR. Cg-Lep^*ob*/ob^ (*ob/ob*) mice. The male *db/db* mice were 8, 16, and 24 weeks^5,7,12^, while male *ob/ob* mice were 5 and 13 weeks of age^6^. A female *ob/ob* dataset was also available from 26 weeks old BTBR.Cg-Lep^*ob/ob*^ mice^13^. Each dataset was originally generated using sciatic nerve samples on the Affymetrix Mouse Genome 430 2.0 array platform. As previously reported, each model displayed the features typical of diabetes by the termination of their respective studies as well as the hallmarks of DPN^5,6,13^. The human sural nerve data used was generated using the Affymetrix Human Genome U133 Plus 2.0 array platform^7^. As previously reported these samples were evaluated for features of DPN and were separated into progressive and non-progressive groups based on the myelinated fibre density lost over a 52 week period^7^.

### Study design

**Figure 1:**
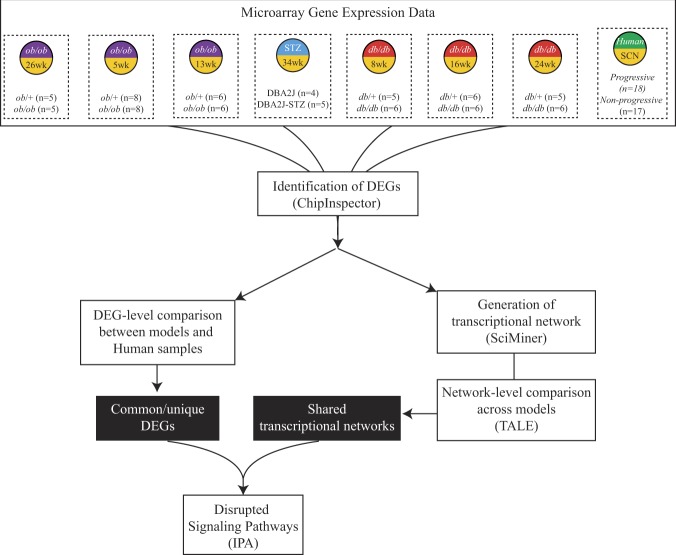
Model and Network DEG Comparison Workflow. Previously published microarray datasets were reanalysed using ChipInspector to identify DEGs at various time points from murine models of type 1 diabetes (STZ), type 2 diabetes (*db*/*db* and *ob*/*ob*), and human patients. DEG datasets from all murine models were generated by comparing diabetic to healthy control mice. The human samples were grouped into progressive and non-progressive groups to determine the DEG dataset used. All DEG datasets were compared in order to find the DEGs shared across models and stages of DPN. These shared DEGs were analysed using IPA to identify possible disrupted signalling pathways. Seven pairwise comparisons were performed to determine the shared networks between each murine dataset with our human dataset. These DEGs were then analysed using IPA to identify possible disrupted signalling pathways.

Figure 1 illustrates the overall workflow of the current study, designed to reanalyse these microarray data from sciatic nerve (SCN) samples taken from our T1DM and T2DM murine models when compared to controls and the human microarray data of sural nerve biopsies obtained from patients with progressive and non-progressive DPN. The original datasets were separated into these groups (either diabetic vs healthy or progressive vs non-progressive) and were compared using ChipInspector (Genomatix Software GMBH, http://www.genomatix.de) to identify the differentially expressed genes (DEGs) between groups. The DEG lists identified from each murine model were converted to the human orthologue equivalent when possible using the Genomatix annotation orthologue database. These DEG lists of human gene IDs were then compared across datasets to identify the conserved DEGs between the different murine models and human. Each DEG set was also analysed using Ingenuity Pathway Analysis (IPA; http://www.ingenuity.com) from Qiagen (Hilden, Germany) and the identified pathways were compared across models and species to identify the commonly disrupted pathways. To identify gene networks common between models, networks were generated from these DEG datasets using SciMiner^14^. The networks generated were analysed for common network nodes and disrupted pathways using IPA.

### Transcriptomic profiling

Transcriptomic data generated from Affymetrix GeneChips were analysed using BioConductor (https://www.bioconductor.org/) and their Affymetrix QC packing in R (http://www.arrayanalysis.org/). All data passing our quality threshold were then analysed using ChipInspector and up-to-date gene annotation. Expression signals from the microarray image files were analysed at the single probe level. Significant transcripts were defined using a minimum of five significant probes and a false discovery rate of <1% by the Significance Analysis of Microarrays algorithm using exhaustive comparisons between control and diabetic mice. Eight datasets of DEGs were generated based on significant transcripts between control and diabetic groups for each murine model (8, 16, and 24 week *db*/*db*; 5, 13, 26 week *ob*/*ob*; and 34 week STZ) as well as between progressive and non-progressive groups for the human dataset^5–7,9,10,12,13^.

### Diabetes- and Age-Comparisons of DEGs Sets

The eight DEG sets were compared to find shared DEGs between species, stages of neuropathy, and diabetes type at a gene and network level. Each dataset was assessed in previous publications for characteristics of DPN and defined as a specific stage of DPN^5–7,9,12,13^. All DEG sets were examined for overlap to identify the conserved pathways or genes responsible for the underlying cause of DPN in the different murine and human samples.

### Transcriptional network comparison

Each DEG set was used to generate transcriptional networks based on gene-gene associations that were identified from biomedical literature using SciMiner^14^. SciMiner is our in-house literature mining system that analyses over 24 million abstracts in PubMed to automatically extract potential gene-gene interactions based on their co-occurrence at the sentence level. The network for each of the eight DEG sets were constructed individually following mouse gene to human gene orthologue conversion according to the Geomatics annotation orthologue database. Cytoscape (http://www.cytoscape.org)^15^, an open-source platform for visualizing complex networks, was used to visualize SciMiner-generated transcriptional networks.

Each mouse DEG network was compared to the human DEG network in a pairwise manner, using a Tool for Approximate Subgraph Matching of Large Queries Efficiently (TALE)^16^. TALE compares network structure and extracts overlapping conserved relationships between two networks. The mismatch parameter used allows 10% mismatch when generating seed gene nodes and extended networks as in our previous studies^17,18^. Conserved nodes across networks were then examined and analysed for overlapping pathways using IPA.

### Functional enrichment analysis

IPA was used to identify enriched pathways within each of the eight DEG sets as well as the seven TALE generated network comparison datasets (comparing each mouse DEG set to our human DEG set). These pathways for the eight DEG sets were compared to identify conserved disrupted pathways that may indicate an underlying cause to DPN. The disrupted pathways observed in our TALE datasets were also compared in order to identify a possible central pathway based on nodes and subnetworks generated from our transcriptional network comparison. The background list of genes used for this analysis was the Affymetrix Human Genome U133 Plus 2.0 Array.

### Construction of Merged Human-Mouse-Conserved Transcriptional Network and Network Centrality Analysis

Eight TALE networks were combined using the merge function in Cytoscape. Edges supported by less than three citations according to SciMiner were removed from the network. The merged human-mouse-conserved transcriptional network was examined to identify the most central genes in the network. Using package ‘*sna*’, tools for social network analysis, in R (https://CRAN.R-project.org/package=sna), four centrality metrics (degree, eigenvector, closeness, and betweenness) were computed to identify the most important nodes (*i*.*e*., genes) in the merged transcriptional network. These four different centrality metrics measure different aspects of node characteristics^19,20^.

Briefly, the degree centrality is the number of nodes that are its first neighbours (*i*.*e*., directly connected to the given node). The more connections a node has, the more central it is, based on degree centrality. In eigenvector centrality, a node contributes to the centrality of another node proportionally to its own centrality. A node is more central, if it is connected to many central nodes. The other two metrics (closeness and betweenness) are dependent on the position of a node in the network. Closeness centrality is based on the distance of a node to the other nodes in the network. The closer a node is to the other nodes, the more important it is considered to be. Betweenness centrality is based on the number of shortest paths connecting two nodes that pass over the given node. A node is more central, if it acts like a bridge in the network, *i*.*e*., lies on many shortest paths. In the current study, we defined the top 10 or 50 most central genes, belonging to the ranks of the genes using each metric. These gene sets were further examined for their enriched biological functions using IPA.

The validity of the central genes was examined by comparing against the average centrality scores from randomly generated transcriptional networks. We generated 1,000 gene sets for each of the eight DEG sets containing the same number of genes randomly selected from all the genes available on the microarray platform. These random gene sets were processed in the same exact way as the real datasets, which resulted in 1,000 merged shared networks. The four centrality scores were measured for each gene in the networks and were used as the background distribution for a Z-test of the centrality scores from the real data.

## Results

### Identification of changes in gene expression

Gene expression profiles were generated using eight published datasets from sciatic nerve samples from both T1DM and T2DM murine models with DPN as well as human sural nerves from patients with T1DM and T2DM^5–7,9,10,12,13^. Metabolic and neuropathy phenotyping on all animal models as well as human subjects are summarized in Supplementary Table 2 based on the published reports. Briefly, T2DM mouse models (*ob*/*ob* and *db*/*db*) were significantly heavier and displayed higher levels of fasting glucose levels and glycosylated hemoglobin, when compared with age-matched non-diabetic controls (*ob*/+ and *db*/+, respectively). Triglyceride levels were significantly increased in T2DM mice. STZ-induced T1DM mice had significantly reduced body weight and higher fasting blood glucose and glycosylated hemoglobin levels relative to non-diabetic controls. Motor and sensory nerve conduction velocities (NCVs) were significantly lower in diabetic mice at all stages of diabetes. Intra-epidermal nerve fibre density (IENFD) was significantly decreased in *ob*/*ob* mice at 9 and 13 weeks compared with age-matched control littermates. Similar changes were observed in the *db*/*db* mouse model at 16 and 24 weeks of age. Both IENFD loss and reduced NCVs confirm the development of DPN in murine models of disease^21^. In the human subjects, there was a change in myelin fibre density (MFD) over the course of the 52-week study between subjects with progressive versus non-progressive neuropathy^10^. However, other factors, including gender, age, insulin treatment, triglyceride levels, glycosylated haemoglobin and body mass index (BMI), were not significantly different between the two groups.

**Figure 2:**
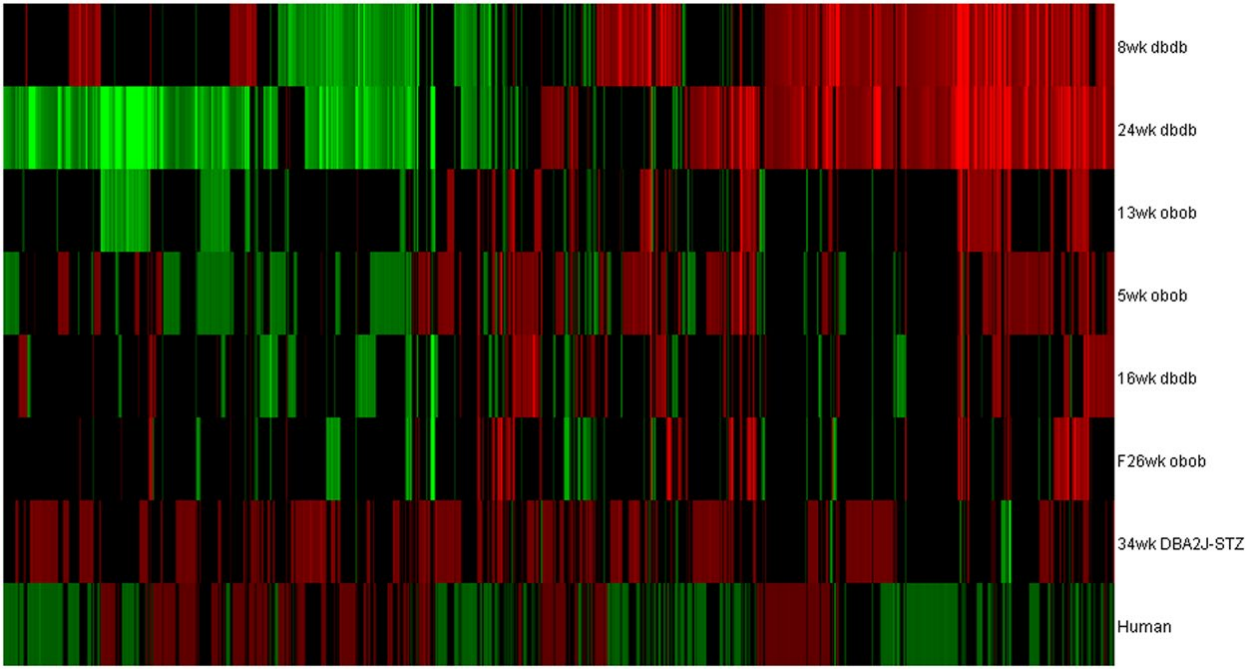
DEG patterns by DPN model. Over 11,000 genes were identified and at least 2,100 were shared across a minimum of 3 datasets. This heat map shows the pattern of distribution for DEGs across models to display how similar or different each model used in this analysis appeared to be on a transcription level.

ChipInspector was used to reanalyse each dataset and identified between 438–5,757 DEGs within each dataset (Table 1). Gene expression profiles were based on a healthy control vs DPN comparison for each murine model while the human sural nerve comparison was based on comparing patients with progressive versus non-progressive DPN^10^. Across all eight datasets, over 11,000 genes were identified with at least 2,100 being shared across at least 3 datasets. Most murine models displayed a similar expression pattern while our T1DM model (STZ-treated) showed a distinct pattern (Fig. 2). Some of the most common genes identified across these models include interleukin 1 receptor antagonist (IL1RN)^22,23^, complement component 3a receptor 1 (C3AR1), macrophage scavenger receptor 1 (MSR1)^24,25^, and matrix metallopeptidase 12 (MMP12)^26^, which have been implicated in the pathogenesis of DPN. Supplementary Fig. 1 illustrates the enrichment levels of the most frequently enriched pathways identified in at least five DEG sets in these datasets by IPA. These pathways include many genes related to lipid metabolism, extracellular matrix homeostasis, and immune signalling, which have all been implicated in the pathogenesis of DPN^6,7,9,11–13,17,27,28^.

**Table 1:** Dataset Summary.

Dataset	Number of Control Samples	Number of Diabetic Samples	DEGs identified by ChipInspector
8wk *db*/*db*	6	5	2,955
16wk *db*/*db*	6	6	871
24wk *db*/*db*	6	6	5,068
5wk *ob*/*ob*	8	8	2,096
13wk *ob*/*ob*	6	6	723
Female 26wk *ob*/*ob*	5	5	482
34wk DBA2J-STZ	4	5	3,022
Human sural nerve	17 non- progressive	18 progressive	5,757

The number of samples for both control and diabetic samples within each dataset is represented in the 2^nd^ and 3^rd^ column. The human dataset rather than being healthy versus diabetic samples were grouped into non-progressive and progressive groups based on myelin fibre density loss. The amount of DEGs identified by ChipInspector ranged from 482 to 5,757 for each dataset.

### Transcriptional network analysis

Our network analysis refines the datasets by examining the connections between identified DEGs to allow prioritization of possibly centrally influential genes or pathways. Prior to pathway analysis with IPA, transcriptional networks are generated based on gene-gene associations, among DEGs, that are identified for each dataset using SciMiner^14^. These networks were then compared in a pairwise manner between murine and human datasets using TALE with a 10% mismatch parameter to limit the shared network^16^. Networks ranging from 46–396 genes were identified as shared between each murine set of DEGs and the progressive human set of DEGs (Supplementary Table 1; Supplementary Fig. 2). The top network DEGs identified as shared across datasets within the compared networks are presented in Supplementary Fig. 3. Among these shared DEGs there is a large number of immune factor genes such as CD44, Interleukin 1 receptor antagonist, and macrophage scavenger receptor 1.

Other notable genes identified in this shared network include dipeptidyl peptidase 4 (DPP4), a serine peptidase that modulates the levels of incretin hormones, major regulators of glucose homeostasis^29^. The antidiabetic action of DPP4 inhibition has been associated with a partial amelioration of NCV deficit in T1DM rats as well as a reduction in nerve fiber loss^30,31^. The identification of the immune genes previously described as well as other target genes associated with DPN suggests that our network approach can successfully narrow the focus of transcriptomic data.

**Figure 3:**
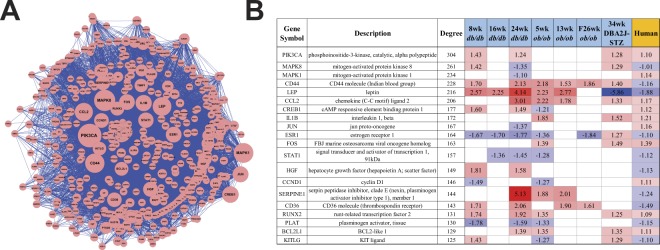
Highly connected DEGs across TALE networks. (**A**) TALE networks were combined using the merge network feature in Cytoscape. Node size is based on the degree of connections and organized as a radial tree. (**B)** This table shows the fold changes of the most highly connected genes in each dataset with red colouring being an increased fold change and blue being a decreased fold change. A total of 688 genes were included in the network with the degree of connections between genes ranging from 304 and 1. Each connection between genes were supported by a minimum of 3 citations as defined by SciMiner.

Cytoscape was then used to visualize each shared TALE network. Networks were merged using the merge network feature in Cytoscape in order to identify the most connected DEGs (Fig. 3). Each individual shared network based on the TALE comparison between the human dataset and each murine dataset can be found in the supplemental material (Supplemental Fig. 2). The top five most highly connected genes based on degrees within the merged network were PIK3CA, MPAK8, CD44, MAPK1, and CREB. These most connected genes are not biased by the amount of dataset in which they appear, but instead depend on the connections that each node has within the network. The majority of highly connected genes do appear in four or more datasets and all genes are observed in our human data.

**Figure 4:**
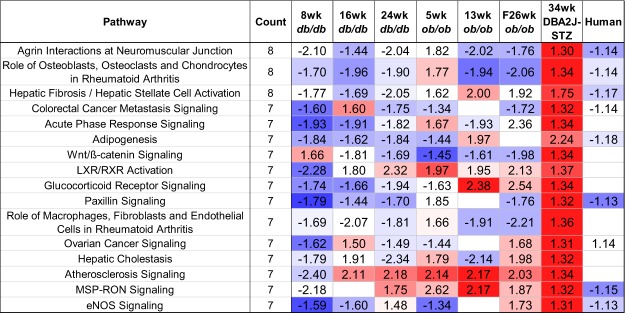
Disrupted Pathways Based on TALE DEGs. The most frequently perturbed pathways within each shared network are represented in the table. The cell value indicates the average change in fold change for the genes involved in this pathway while the colour indicates the overall direction of the genes. Red indicates that more genes involved in the pathway have increased expression while blue indicates the genes involved have decreased expression values. The most common theme among these pathways are inflammation with multiple interleukin signalling pathway as well as some autoimmune pathways commonly found in rheumatoid arthritis.

The genes in the shared transcriptional networks between human and each murine model were analysed for pathway enrichment using IPA. Over 380 pathways were found to be significantly enriched and the top pathways shared across each network are shown in Fig. 4, which displays a fold change based on the average fold change of the pathway genes identified by IPA. The directionality is indicated by colour (with red being an increase and blue being a decrease) based on the percentage of genes involved that have increased or decreased expression levels. These pathways were often directionally similar to our human dataset, unlike the originally described DEGs. The T1DM STZ model used in our study does display consistent up-regulation of the subset of enriched pathways identified as shared across models in Fig. 4. The top pathways shared across each network included LXR/RXR activation, agrin interactions at neuromuscular junctions, hepatic fibrosis, and role of osteoblasts, osteoclasts, and chondrocytes in rheumatoid arthritis. The common themes that underlie these enriched pathways are lipid metabolism, extracellular matrix, and disrupted inflammation.

### Merged transcriptional network and centrality analysis

Seven human and murine shared networks were merged into a single network, consisting of 688 genes that interacted with up to 304 other genes in the network (Fig. 3A). Using the *sna* package in R, this merged network was analysed to identify the most central and influential genes within the network. The four measures of centrality used included closeness, betweenness, degree, and eigenvector values. Table 2 lists the 14 most central genes that are ranked among top 10 in each centrality measure (Table 2). While these genes exhibit overlap with Fig. 3B, they also represent a more thorough and analytical measure of gene influence within the network while the previous figure assists in clarifying the complex merged network. These genes include interleukin 1 beta (IL1B), hepatocyte growth factor (HGF), c-c motif chemokine ligand 2 (CCL2), CD36, FOS, and JUN. While transcription factors such as FOS and JUN are likely to be central to a network as regulators of many genes, the inclusion of cytokine and chemokines such as IL1B, HGF, and CCL2 further supports the involvement of the immune system in DPN^27,32–34^.

**Table 2:** Centrality Analysis Gene Results.

Symbol	Description	Degree (p-value)	Closeness (p-value)	Betweenness (p-value)	Eigenvector (p-value)
PIK3CA	phosphoinositide-3-kinase, catalytic, alpha polypeptide	406 (p = 1.8E−08)	0.00081 (p = 6.4E−01)	43246.5 (p = 0.0E + 00)	0.16 (p = 2.9E−03)
MAPK8	mitogen-activated protein kinase 8	372 (p = 3.8E−07)	0.00078 (p = 6.6E−01)	30937.8 (p = 0.0E + 00)	0.15 (p = 6.3E−03)
CD44	CD44 molecule (Indian blood group)	349 (p = 1.3E−08)	0.00075 (p = 6.6E−01)	32129.4 (p = 0.0E + 00)	0.13 (p = 7.3E−03)
MAPK1	mitogen-activated protein kinase 1	280 (p = 3.5E−03)	0.00074 (p = 6.5E−01)	19719.2 (p = 1.7E−03)	0.13 (p = 3.9E−02)
CREB1	cAMP responsive element binding protein 1	283 (p = 1.2E−08)	0.00073 (p = 6.4E−01)	15524.7 (p = 0.0E + 00)	0.12 (p = 3.0E−03)
LEP	leptin	301 (p = 8.7E−10)	0.00072 (p = 6.6E−01)	22639.7 (p = 0.0E + 00)	0.12 (p = 5.6E−03)
CCL2	chemokine (C-C motif) ligand 2	276 (p = 3.3E−03)	0.00071 (p = 6.9E−01)	13595.3 (p = 4.5E−03)	0.12 (p = 1.0E−01)
JUN	jun proto-oncogene	232 (p = 5.9E−03)	0.00071 (p = 6.7E−01)	11905.8 (p = 1.2E−02)	0.12 (p = 4.3E−02)
ESR1	estrogen receptor 1	269 (p = 4.7E−09)	0.00071 (p = 6.6E−01)	17433.2 (p = 0.0E + 00)	0.11 (p = 5.8E−03)
FOS	FBJ murine osteosarcoma viral oncogene homolog	229 (p = 3.1E−03)	0.00070 (p = 6.4E−01)	21885.2 (p = 2.5E−07)	0.10 (p = 7.4E−02)
CD36	CD36 molecule (thrombospondin receptor)	247 (p = 5.0E−07)	0.00070 (p = 6.5E−01)	11865.1 (p = 6.5E−13)	0.11 (p = 7.2E−03)
IL1B	interleukin 1, beta	224 (p = 6.5E−03)	0.00070 (p = 6.6E−01)	12492.5 (p = 3.4E−03)	0.09 (p = 1.6E−02)
HGF	hepatocyte growth factor (hepapoietin A; scatter factor)	213 (p = 9.4E−06)	0.00069 (p = 6.6E−01)	8254.8 (p = 2.6E−07)	0.12 (p = 4.6E−03)

Centrality analysis was conducted using the Cytoscape plug-in CentiScaPe and four centrality metrics (degree, eigenvector, closeness, and betweenness) to identify the most important nodes (*i*.*e*., genes) in the merged transcriptional network. The top 10 ranked genes in each perspective centrality metric is included in the table and indicate the most influential genes within the network. The centrality scores of each node were compared against the background distribution of centrality scores that were obtained from randomly generated 1,000 random merged networks. P-values were calculated using z-test to examine the significant difference between the real and random networks.

The 14 most central genes were also used as input to IPA in order to identify enriched pathways represented by these influential genes. The top 20 canonical pathways are shown in Table 3 with −log_10_(p-value) as a measure of significance; the ratio represents the proportion of the 14 central genes to all the genes involved in the canonical pathway. Ratio values in this case are expected to be low since our input gene list was only the 14 genes identified as most central within the network. The most significant pathways include HMGB1 signalling and Glucocorticoid receptor signalling.

**Table 3:** Top 20 IPA Canonical Pathways Based on the Most Central Genes.

Ingenuity Canonical Pathways	−log_10_(p-value)	Genes	Ratio
HMGB1 Signalling	14.2	FOS, PIK3CA, JUN, CCL2, MAPK1, MAPK8, IL1B, PLAT	0.06
Glucocorticoid Receptor Signalling	13.6	FOS, PIK3CA, JUN, CCL2, MAPK1, CREB1, MAPK8, IL1B, ESR1	0.03
GDNF Family Ligand-Receptor Interactions	11.3	FOS, PIK3CA, JUN, MAPK1, CREB1, MAPK8	0.08
Neurotrophin/TRK Signalling	11.3	FOS, PIK3CA, JUN, MAPK1, CREB1, MAPK8	0.08
Estrogen-Dependent Breast Cancer Signalling	11.2	FOS, PIK3CA, JUN, MAPK1, CREB1, ESR1	0.08
LPS-stimulated MAPK Signalling	11	FOS, PIK3CA, JUN, MAPK1, CREB1, MAPK8	0.07
HGF Signalling	10.2	FOS, PIK3CA, JUN, MAPK1, HGF, MAPK8	0.05
Renin-Angiotensin Signalling	10.1	FOS, PIK3CA, JUN, CCL2, MAPK1, MAPK8	0.05
IL-6 Signalling	9.92	FOS, PIK3CA, JUN, MAPK1, MAPK8, IL1B	0.05
Aryl Hydrocarbon Receptor Signalling	9.66	FOS, JUN, MAPK1, MAPK8, IL1B, ESR1	0.04
Role of Macrophages, Fibroblasts and Endothelial Cells in Rheumatoid Arthritis	9.38	FOS, PIK3CA, JUN, CCL2, MAPK1, CREB1, IL1B	0.02
IL-2 Signalling	9.37	FOS, PIK3CA, JUN, MAPK1, MAPK8	0.08
UVB-Induced MAPK Signalling	9.3	FOS, PIK3CA, JUN, MAPK1, MAPK8	0.08
IL-10 Signalling	9.23	FOS, JUN, MAPK1, MAPK8, IL1B	0.07
EGF Signalling	9.23	FOS, PIK3CA, JUN, MAPK1, MAPK8	0.07
Acute Phase Response Signalling	9.17	FOS, PIK3CA, JUN, MAPK1, MAPK8, IL1B	0.04
Chemokine Signalling	9.14	FOS, JUN, CCL2, MAPK1, MAPK8	0.07
Toll-like Receptor Signalling	9.05	FOS, JUN, MAPK1, MAPK8, IL1B	0.07
CD40 Signalling	8.93	FOS, PIK3CA, JUN, MAPK1, MAPK8	0.06
Dendritic Cell Maturation	8.86	PIK3CA, LEP, MAPK1, CREB1, MAPK8, IL1B	0.03

The 14 genes identified by CentiScaPe to be the most central genes within the merged network based on the four centrality measures were used as input for IPA to analyse pathway enrichment. This table represents the enriched pathways based on these genes with −log_10_(p-value) as a significance measure and the ratio as the proportion of significant DEGs measured over the total genes within the pathway. HMGB1 signalling, Glucocorticoid receptor signalling, as well as the various interleukin pathways indicate disrupted inflammation as a central influence within the cross-species shared network. The ratio represents the proportion of the 14 central genes to all the genes involved in the canonical pathway.

**Figure 5:**
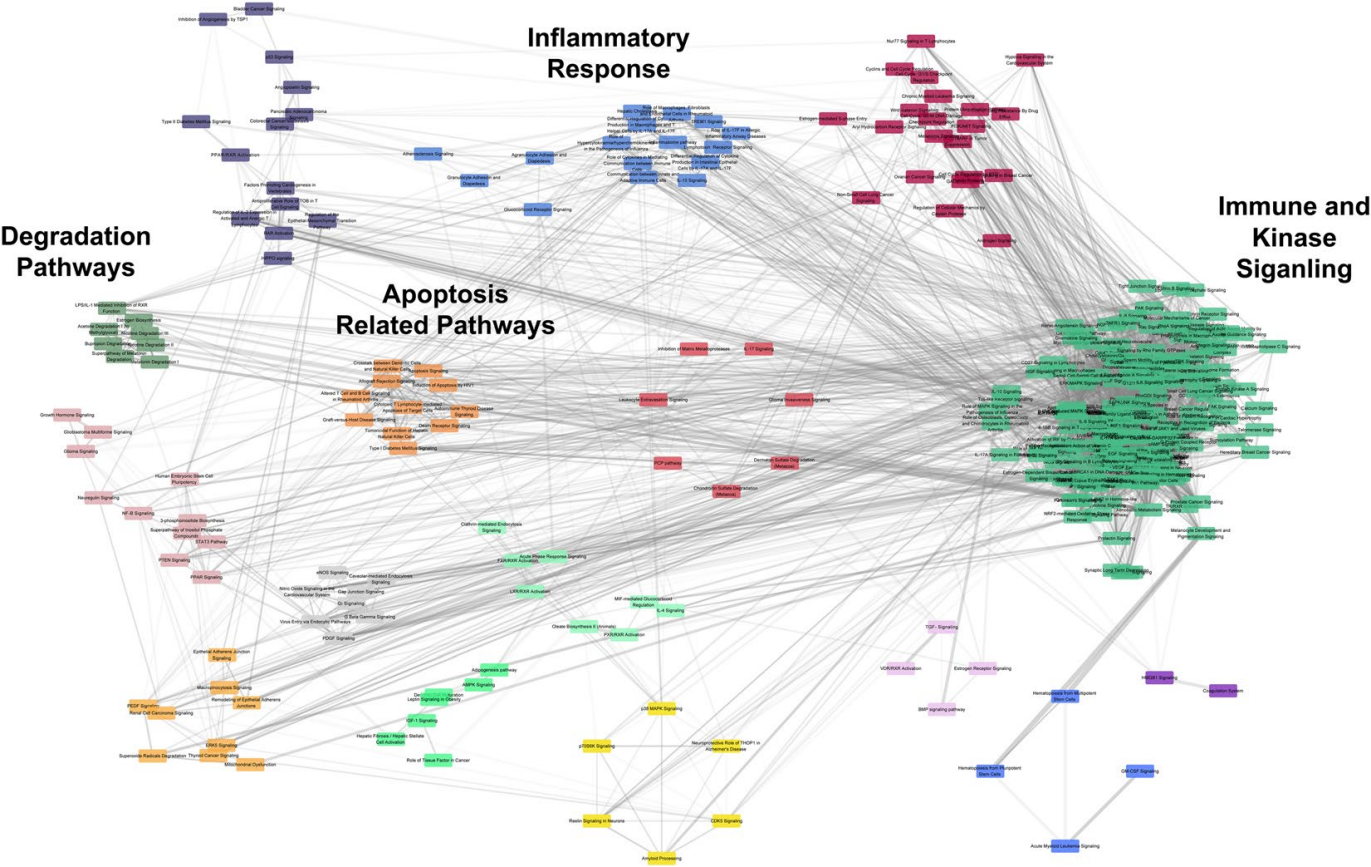
IPA Enriched Pathway Clustering. The pathways found to be enriched by IPA based on the top 64 most central genes, belonging to the top 50 in at least one centrality measure, within the merged network. 272 canonical pathways determined significantly enriched by IPA were examined for their similarity in terms of gene content and shared directionality among the pathways. Edges, connections between pathways, are only included if their similarity scores were among the top 25%. InfoMap^35^, a network clustering package in R, was used to identify clusters, which are represented in different colours. These clusters shared common functional themes, which are noted in the figure. The largest cluster of canonical pathways were associated with immune response and inflammation (in green). Colours of the node denote the clusters identified by InfoMap.

We further extended our pathway enrichment analysis to the 64 most central genes, belonging to the top 50 in at least one centrality measure. A total of 272 canonical pathways were identified to be significantly enriched (Supplementary Table 3). Based on gene overlap and shared directionality among pairs of these pathways, we constructed a contextual similarity network in Fig. 5, where edges from the top 25% similarity scores are included. InfoMap^35^, a network clustering algorithm, was used to identify sub-networks or clusters that are highly interconnected. These clusters shared common functional themes, and the largest cluster of canonical pathways was associated with immune response and inflammation (in dark mint in Fig. 5).

## Discussion

In the current study, we compared transcriptomic changes in peripheral nerves isolated from humans and mouse models of T1DM and T2DM at various stages of DPN to identify potential molecular pathways contributing to disease. We previously examined these changes during the development of DPN in both mouse models and human patients^6,7,9,10^ and determined critical genes and pathways that play an important role in DPN. However, no systematic comparison of these transcriptomics datasets has been made. In the current study, using our published datasets, transcriptomic changes were compared in multiple mouse models of diabetes at different stages of the disease as well as in human subjects with DPN. Changes in one human and seven murine microarray datasets - at both the gene and pathway level – were examined using a single unified analysis pipeline to identify common pathways involved in the development of DPN. In total, we identified over 380 pathways that were enriched across all datasets, providing new insights into DPN pathogenesis.

Many of the pathways that were dysregulated in our previous reports were similarly dysregulated when compared across the seven murine models and human samples, including pathways associated with immune system, cellular development and cellular survival^5,7,10,17,28^. In addition, we show that key pathways governing lipid metabolism (LXR/RXR, adipogenesis and PIK3CA) and extracellular matrix homeostasis (chondrocytes, paxillin, and fibrosis), that have been highlighted in our previous study, were also altered in the current setting^7^. Consistent with previous reports, transcriptional changes associated with estrogen signalling were observed, as sex-specific risk levels for diabetic complications have been well documented in human^36^ and mouse^13,37^ models.

In agreement with our previous studies^5–7,11–13,28^, we observed transcriptomic and functional pathway changes in multiple immune-related pathways in all of the datasets. In particular, our results indicate that key pathways involved in the control of immune and inflammatory functions were upregulated during DPN, including NF-κB and JAK/STAT pathways. In fact, these pathways are activated in the DRGs of diabetic rats and have been associated with nerve injury in diabetes^17,38,39^. Our findings support targeting these pathways in murine models of diabetes to understand their pathophysiological roles in DPN^18,40^. We found that both pro- and anti-inflammatory cytokine pathways were dysregulated across datasets: IL-2, IL-6, and IL-10 as well as chemokines were all altered in DPN. Studies have shown that cytokines and chemokines not only promote existing inflammatory and immune responses, but also induce oxidative and nitrosative stress, further exacerbating cellular injury in experimental models of DPN^41^. In fact, during inflammation, both pro- and anti-inflammatory pathways are often simultaneously engaged as a disease process such as DPN transitions from active to chronic inflammation. For instance, neuronal repair is initiated by neutrophils^42^ and driven by macrophages^43^ under specific environmental conditions that are anti-inflammatory; however, as the disease process progresses, the introduction of pro-inflammatory signals overrides the anti-inflammatory response, resulting in tissue destruction. This concept is established in neurodegenerative disorders such as amyotrophic lateral sclerosis^44^, and our data strongly suggest a similar process is occurring in DPN.

The importance of the immune system is further highlighted by the fact that many of the pathways we observed dysregulated across all murine models and human samples, including those involved with transcription, cellular development, and lipid metabolism are also involved in the immune response. We show that HMGB1 signalling, which is centrally involved in the regulation of gene transcription, is one of the most highly dysregulated canonical pathways. However, HMGB1 is also secreted by macrophages and damaged cells and mediates systemic inflammation^45^ by signalling through receptors such as the Receptor for Advanced Glycation End-products (RAGE) and Toll-Like receptors (TLRs), both of which have been implicated in obesity-driven inflammation and DPN^46–49^. This signalling cascade may drive NF-κB pathway that further exacerbates the inflammatory response and increases tissue damage.

Moreover, disrupted lipid metabolism is associated with inflammation. PPAR-γ, a major regulator of lipid and glucose metabolism, is altered in murine models as well as in human and has been previously implicated as a common factor in both DPN and diabetic nephropathy^5,9^. Yet, PPAR-γ can also control inflammation in macrophages and dendritic cells^50,51^. Besides its established role in inflammation, the LXR/RXR system has emerged as a key regulator of cholesterol, fatty acid and glucose homeostasis, and neuroprotection^52–54^. Additionally, LXR/RXR has been increasingly shown to play an important role in diabetic complications^54–57^. Interestingly, LXR/RXR is observed as a shared pathway across murine models^51,58^. To a further extent, our results show an initial downregulation of the LXR/RXR pathway in the *db*/*db* mouse model after 8 weeks of diabetes and an upregulation at late stages of the disease. In contrast, LXR/RXR expression was upregulated in the *ob*/*ob* mouse model throughout the duration of diabetes and after 34 weeks of diabetes in STZ-induced T1DM mice, while no data were reported at earlier time points for T1DM mice. Collectively, our findings point toward a potential involvement of the LXR/RXR pathway in DPN. Taken together, our data further support a pivotal role of the immune system in DPN, though it is unclear from the current data to what extent this involvement is a cause or consequence of neuronal damage. We are currently addressing this question in both experimental^27^ and clinical settings (https://clinicaltrials.gov/show/NCT02936843).

On a different note, while numerous pathways were dysregulated across datasets, the direction of transcriptional change was rarely universal. For many pathways, we found that mouse models of DPN and human tissue transcription were differentially expressed in opposite directions; whereas pathways in mice were upregulated, they were frequently downregulated in human patients. Similar cross-species discrepancy in gene expression direction was also observed in diabetic nephropathy between human and murine models^18^. There are several potential explanations for these discrepancies. The first is that the kinetics of murine and human DPN progression are different; we observed transcriptomic changes in mouse models of DPN up to 24 weeks (T2DM) and 34 weeks (T1DM), but it is unlikely these mice completely represent the advanced stages of DPN encountered in human after decades of disease. Tissue sources may also account for these differences, as the human dataset was obtained from sural nerves while mouse data were collected from sciatic nerves. We are currently exploring discordant transcriptomic dysregulation in the sciatic and sural nerves of mouse models, as a similar effect may be occurring here.

Alternatively, the nature of the controls in each species may have influenced the results. Whereas changes observed in the murine models are a result of a diabetic versus non-diabetic comparison, human transcriptomic data are a result of comparisons between progressive and non-progressive DPN. We recently conducted a transcriptional network analysis of DPN progression using the same *db/db* microarray data at 8, 16, and 24 weeks^12^ used in the current study. This study identified various DPN progression-associated genes and pathways, which were overlapping with those identified in the present study, such as TLR signalling, dendritic cell maturation, LXR/RXR activation, and various cytokine pathways. Furthermore, O’Brien *et al*.’s original publication on the *ob/ob* mouse model data at 5 and 13 weeks^6^ included a similar comparative analysis result. In this study, inflammatory mechanisms were found to have a critical role in early development and progression of DPN, and genes related to inflammation, immune response, and chemotaxis were highly enriched at 5 weeks rather than 13 weeks. Both studies identified MMP12 as the most significantly up-regulated gene across different time points, which was also found to be shared between human and murine DPN in the current study, suggesting its potentially critical role in the progression of DPN in both the experimental and clinical settings. Finally, in order to compare changes between species, an orthologue conversion was also used to convert murine gene identifiers to their human equivalent genes; as such it is possible that some murine genes were not captured in the current analyses.

We also found that many pathways that were downregulated or a mix of up/down regulation in T2DM-driven DPN were strongly upregulated in the dataset from the T1DM STZ mouse model. To validate these data, we re-examined the outlier parameters in the dataset as well as the background levels of each microarray; in both cases the exclusion criteria remained unmet (data not shown). The STZ dataset was held to the same criteria as all other murine models which allows for a unified comparison across models and species, yet we still found that most of the detected pathways were highly upregulated in T1DM. These data suggest that while similar molecular pathways are involved in DPN progression, the utilization of these pathways may be very different in T1DM and T2DM. This is supported by our previous study examining transcriptional changes in diabetic neuropathy and nephropathy in both T1DM and T2DM mouse models; we found that while there was a high transcriptional concordance in diabetic nephropathy, DPN-associated pathways were often highly discordant^17^. Together these data suggest that key molecular pathways are commonly involved in DPN but that they may be differentially regulated in T1DM and T2DM. This concept is further supported by our reports in man, where a systemic review of clinical trials strongly suggest different pathogenic mechanisms underlying DPN in T1DM versus T2DM^59^.

## Conclusions

In summary, our transcriptional network-based approach, integrating multiple bioinformatics analyses, identified DPN-associated pathways that are highly conserved across multiple murine models and human. Many of the pathways identified highlight the importance of the immune system through cytokine and chemokine signalling as well as the observed dysregulated pathways associated with transcription, cellular development, and lipid metabolism are all involved in the immune response. The observed conserved pathways are likely the key responses in DPN and provide new therapeutic targets for the potential treatment of DPN, a disorder that remains without a drug intervention to date.

## Electronic supplementary material

Supplementary Materials

## Data Availability

The microarray raw data used to support the findings of this study were gathered from the Diabetic Neuropathy Microarray Knowledge-Base (DNMKB; http://hurlab.med.und.edu/DNMKB/) and previous publications. These prior studies (and datasets) are cited at relevant places within the text as refs^5–13^.
