# Supplementary material for: Conserved Transcriptional Signatures in Human and Murine Diabetic Peripheral Neuropathy
Source: Sci Rep. 2018 Dec 5;8:17678. doi: 10.1038/s41598-018-36098-5 (PMC6281650; doi:10.1038/s41598-018-36098-5)
Supplement: Supplementary file 1 — Supplementary Materials [file 41598_2018_36098_MOESM1_ESM.docx]

**Supplementary Materials**

Conserved Transcriptional Signatures in Human and Murine Diabetic Peripheral Neuropathy

Running title: Cross-Species Transcriptional Networks in DPN

Brett A. McGregor^1,#^, Stephanie Eid^2,#^, Amy E. Rumora^2^, Benjamin Murdock^2^_,_ Kai Guo^1^, Guillermo de Anda-Jauregui^1^, James E. Porter^1^, Eva L. Feldman^2,Φ^, and Junguk Hur^1,Φ^

^1^Department of Biomedical Sciences, University of North Dakota School of Medicine and Health Sciences, Grand Forks, North Dakota 58202, USA

^2^Department of Neurology, University of Michigan, Ann Arbor, Michigan 48109, USA

# Equal contribution.

**^Φ^** Corresponding authors:

Junguk Hur, PhD

Assistant Professor

Department of Biomedical Sciences

University of North Dakota, School of Medicine and Health Sciences

1301 North Columbia Rd. Stop 9037

Grand Forks, ND 58202-9037

Phone: (701) 777-6814

Fax: (701) 777-2477

Email: junguk.hur@med.und.edu

Eva L. Feldman, MD, PhD

Russell N. DeJong Professor of Neurology

5017 AATBSRB, 109 Zina Pitcher Place

Ann Arbor, MI 48109, United States

Phone: (734) 763-7274

Fax: (734) 763-7275

Email: efeldman@med.umich.edu

# Supplementary Figures

**Supplementary Figure 1. Most frequently dysregulated canonical pathways in each dataset identified by Ingenuity Pathway Analysis (IPA)**. Each dataset was analyzed using IPA for canonical pathway enrichment then compared to determine the most frequently enriched pathways across datasets. The numbers and color gradient within the table are -log (p values) to represent significant enrichment within each column.

**Supplementary Figure 2. Shared transcriptional networks between murine and human datasets identified by TALE, a graph matching software.** Using a graphical matching software (TALE), each network generated using the murine datasets were examined for overlap with the human gene network. The resulting networks are below with the number in parenthesis representing the total genes within the network. Node size indicates amount of degree relative to the size of the network.

**Supplementary Figure 3. The most commonly dysregulated differentially expressed genes among the shared transcriptional networks.** Shared network genes between each murine dataset and human dataset were identified. The fold change for each dataset is represented by the value in each cell and the color displays the relative change within the table, with red being an increased fold change and blue being a decreased fold change. Many of the represented DEGs are directionally consistent across murine models but not necessarily between the human and murine comparison.


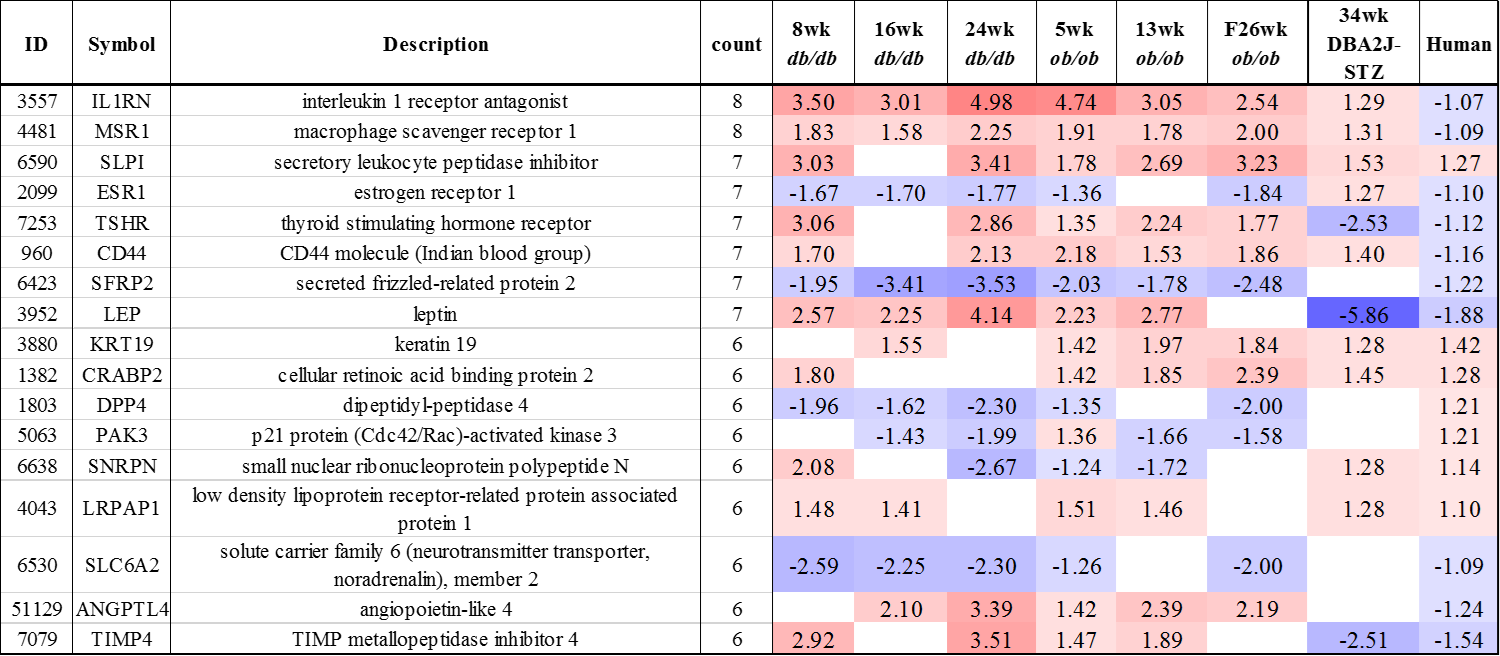


# Supplementary Tables

**Supplementary Table 1. Summary of the microarray datasets.** The number of samples for both control and diabetic samples within each dataset is represented in the 2^nd^ and 3^rd^ column. The human dataset rather than being healthy versus diabetic samples were grouped into non-progressive and progressive groups based on myelin fiber density lost. The amount of DEGs identified by ChipInspector ranged from 482 to 5,757 for each dataset. The superscript numbers in the header row correspond to the reference numbers in the manuscript. wks: weeks.

| **Dataset** | **Number of control samples** | **Number of diabetic samples** | **DEGs identified by ChipInspector** | **Orthologue mapped Human Gene IDs** | **Genes included in the literature-derived network** |
| --- | --- | --- | --- | --- | --- |
| *db/db* 8 wks (M)^12^ | 6 | 5 | 2,955 | 2,782 | 2,194 |
| *db/db* 16 wks (M)^7^ | 6 | 6 | 871 | 794 | 579 |
| *db/db* 24 wks (M)^5^ | 6 | 6 | 5,068 | 4,743 | 3,762 |
| *ob/ob* 5 wks (M)^6^ | 8 | 8 | 2,096 | 1,910 | 1,535 |
| *ob/ob* 13 wks (M)^6^ | 6 | 6 | 723 | 685 | 509 |
| *ob/ob* 26 wks (F)^13^ | 5 | 5 | 482 | 438 | 338 |
| DBA2J-STZ 34 wks (M)^9^ | 4 | 5 | 3,022 | 2,482 | 1,932 |
| Human DPN^10^ | 17 non-progressive | 18 progressive | 5,757 | 5,757 | 4,232 |

**Supplementary Table 2. Metabolic and neuropathy phenotypic measures of the datasets.** The previously published datasets included in this study have all been tested for measurements of metabolic disturbances and neuropathic phenotyping independently. The increase (+) or decrease (-) indicates the changes observed in diabetic animals compared to their respective non-diabetic controls and between the progressive and non-progressive human patients. Not all studies underwent the same testing and measurements not included are indicated by an N/A, while non-significant results (p>0.05) are indicated by N/S. The superscript numbers in the header row correspond to the reference numbers in the manuscript. BMI: body mass index; M: male; F: female. wks: weeks. * indicates that measurements taken at 9 weeks in the published study.

| **Published Datasets Included** | ***db/db* 8 wks (M)^12^** | ***db/db* 16 wks (M)^7^** | ***db/db* 24 wks (M)^5^** | ***ob/ob* 5 wks (M)^6^** | ***ob/ob* 13 wks (M)^6^** | ***ob/ob* 26 wks (F)^13^** | **DBA2J-STZ 34 wks (M)^9^** | **Human DPN^10^** |
| --- | --- | --- | --- | --- | --- | --- | --- | --- |
| **Metabolic measures** | | | | | | | | |
| **Body weight** | ↑ | ↑ | ↑ | ↑ | ↑ | ↑ | ↓ | BMI: N/S |
| **Fasting glucose** | ↑ | ↑ | ↑ | ↑ | ↑ | N/A | ↑ | N/A |
| **Triglycerides** | N/A | ↑ | ↑ | ↑ | ↑ | ↑ | N/A | N/S |
| **% Glycosylated hemoglobin** | ↑ | ↑ | ↑ | ↑ | ↑ | ↑ | ↑ | N/S |
| **Neuropathy phenotype measures** | | | | | | | | |
| **Hindpaw latency** | N/S | ↑ | ↑ | ↑* | N/A | N/A | ↑ | N/A |
| **Motor nerve conduction velocity** | ↓ | ↓ | ↓ | ↓* | ↓ | ↓ | ↓ | N/A |
| **Sensory nerve conduction velocity** | ↓ | ↓ | ↓ | ↓* | ↓ | ↓ | ↓ | N/A |
| **Intra-epidermal nerve fiber density** | N/S | ↓ | ↓ | ↓* | ↓ | ↓ | N/A | N/A |
| **Myelinated fiber density changes over 52 wks** | N/A | N/A | N/A | N/A | N/A | N/A | N/A | ↓ |

**Supplementary Table 3. The most enriched canonical pathways among the most central 14 genes of the human-murine conserved transcriptional network.**  The 14 genes identified by CentiScaPe to be the most central genes within the merged network based on the four centrality measures were used as input for IPA to analyze pathway enrichment. The table below represents the enriched pathways based on these genes with –log(p-value) as a significance measure and the ratio as the amount of genes measured over the total genes within the pathway.

| **Ingenuity Canonical Pathways** | **-log(p-value)** | **Ratio** |
| --- | --- | --- |
| HMGB1 Signaling | 14.2 | 0.06 |
| Glucocorticoid Receptor Signaling | 13.6 | 0.03 |
| GDNF Family Ligand-Receptor Interactions | 11.3 | 0.08 |
| Neurotrophin/TRK Signaling | 11.3 | 0.08 |
| Estrogen-Dependent Breast Cancer Signaling | 11.2 | 0.08 |
| LPS-stimulated MAPK Signaling | 11 | 0.07 |
| HGF Signaling | 10.2 | 0.05 |
| Renin-Angiotensin Signaling | 10.1 | 0.05 |
| IL-6 Signaling | 9.92 | 0.05 |
| Aryl Hydrocarbon Receptor Signaling | 9.66 | 0.04 |
| Role of Macrophages, Fibroblasts and Endothelial Cells in Rheumatoid Arthritis | 9.38 | 0.02 |
| IL-2 Signaling | 9.37 | 0.08 |
| UVB-Induced MAPK Signaling | 9.3 | 0.08 |
| IL-10 Signaling | 9.23 | 0.07 |
| EGF Signaling | 9.23 | 0.07 |
| Acute Phase Response Signaling | 9.17 | 0.04 |
| Chemokine Signaling | 9.14 | 0.07 |
| Toll-like Receptor Signaling | 9.05 | 0.07 |
| CD40 Signaling | 8.93 | 0.06 |
| Dendritic Cell Maturation | 8.86 | 0.03 |
| Renal Cell Carcinoma Signaling | 8.85 | 0.06 |
| IL-17A Signaling in Gastric Cells | 8.82 | 0.16 |
| ILK Signaling | 8.78 | 0.03 |
| IL-17 Signaling | 8.74 | 0.06 |
| FGF Signaling | 8.61 | 0.06 |
| PDGF Signaling | 8.61 | 0.06 |
| ErbB Signaling | 8.43 | 0.05 |
| Cholecystokinin/Gastrin-mediated Signaling | 8.36 | 0.05 |
| RANK Signaling in Osteoclasts | 8.36 | 0.05 |
| UVA-Induced MAPK Signaling | 8.34 | 0.05 |
| Role of Osteoblasts, Osteoclasts and Chondrocytes in Rheumatoid Arthritis | 8.34 | 0.03 |
| Inhibition of Angiogenesis by TSP1 | 8.26 | 0.12 |
| IGF-1 Signaling | 8.25 | 0.05 |
| IL-17A Signaling in Fibroblasts | 8.2 | 0.11 |
| T Cell Receptor Signaling | 8.19 | 0.05 |
| April Mediated Signaling | 8.06 | 0.11 |
| Rac Signaling | 8.04 | 0.04 |
| B Cell Activating Factor Signaling | 7.96 | 0.1 |
| MIF Regulation of Innate Immunity | 7.92 | 0.1 |
| UVC-Induced MAPK Signaling | 7.88 | 0.1 |
| PI3K Signaling in B Lymphocytes | 7.84 | 0.04 |
| GNRH Signaling | 7.82 | 0.04 |
| 14-3-3-mediated Signaling | 7.81 | 0.04 |
| Role of IL-17F in Allergic Inflammatory Airway Diseases | 7.79 | 0.09 |
| PKCÎ¸ Signaling in T Lymphocytes | 7.77 | 0.04 |
| P2Y Purigenic Receptor Signaling Pathway | 7.77 | 0.04 |
| Role of Pattern Recognition Receptors in Recognition of Bacteria and Viruses | 7.69 | 0.04 |
| IL-12 Signaling and Production in Macrophages | 7.55 | 0.03 |
| Relaxin Signaling | 7.46 | 0.03 |
| CXCR4 Signaling | 7.29 | 0.03 |
| PPAR/RXR Activation | 7.12 | 0.03 |
| Thrombopoietin Signaling | 7.1 | 0.06 |
| Role of IL-17A in Arthritis | 7.04 | 0.06 |
| B Cell Receptor Signaling | 7.04 | 0.03 |
| Endothelin-1 Signaling | 7.01 | 0.03 |
| CCR5 Signaling in Macrophages | 6.99 | 0.06 |
| RAR Activation | 6.98 | 0.03 |
| NRF2-mediated Oxidative Stress Response | 6.95 | 0.03 |
| Production of Nitric Oxide and Reactive Oxygen Species in Macrophages | 6.95 | 0.03 |
| IL-8 Signaling | 6.9 | 0.03 |
| ERK/MAPK Signaling | 6.88 | 0.03 |
| BMP signaling pathway | 6.82 | 0.05 |
| Erythropoietin Signaling | 6.75 | 0.05 |
| Regulation of IL-2 Expression in Activated and Anergic T Lymphocytes | 6.75 | 0.05 |
| IL-3 Signaling | 6.67 | 0.05 |
| Prolactin Signaling | 6.67 | 0.05 |
| JAK/Stat Signaling | 6.67 | 0.05 |
| Systemic Lupus Erythematosus Signaling | 6.61 | 0.02 |
| TGF-Î² Signaling | 6.58 | 0.05 |
| Cardiac Hypertrophy Signaling | 6.52 | 0.02 |
| IL-1 Signaling | 6.51 | 0.04 |
| Ceramide Signaling | 6.47 | 0.04 |
| PPAR Signaling | 6.47 | 0.04 |
| Huntington's Disease Signaling | 6.47 | 0.02 |
| Colorectal Cancer Metastasis Signaling | 6.41 | 0.02 |
| Signaling by Rho Family GTPases | 6.41 | 0.02 |
| Corticotropin Releasing Hormone Signaling | 6.16 | 0.04 |
| Neuropathic Pain Signaling In Dorsal Horn Neurons | 6.11 | 0.04 |
| HIF1 Signaling | 6.1 | 0.03 |
| TNFR2 Signaling | 6.08 | 0.1 |
| NGF Signaling | 6.07 | 0.03 |
| 4-1BB Signaling in T Lymphocytes | 5.99 | 0.1 |
| Type II Diabetes Mellitus Signaling | 5.92 | 0.03 |
| G12/13 Signaling | 5.88 | 0.03 |
| CD28 Signaling in T Helper Cells | 5.87 | 0.03 |
| Hepatic Cholestasis | 5.54 | 0.03 |
| Molecular Mechanisms of Cancer | 5.53 | 0.01 |
| iNOS Signaling | 5.52 | 0.07 |
| Cdc42 Signaling | 5.45 | 0.02 |
| TNFR1 Signaling | 5.38 | 0.06 |
| CD27 Signaling in Lymphocytes | 5.3 | 0.06 |
| Hepatic Fibrosis / Hepatic Stellate Cell Activation | 5.29 | 0.02 |
| Role of NFAT in Regulation of the Immune Response | 5.27 | 0.02 |
| AMPK Signaling | 5.24 | 0.02 |
| Leukocyte Extravasation Signaling | 5.06 | 0.02 |
| Agrin Interactions at Neuromuscular Junction | 4.93 | 0.04 |
| ErbB2-ErbB3 Signaling | 4.93 | 0.04 |
| Glioma Invasiveness Signaling | 4.91 | 0.04 |
| Role of MAPK Signaling in the Pathogenesis of Influenza | 4.87 | 0.04 |
| TREM1 Signaling | 4.82 | 0.04 |
| IL-17A Signaling in Airway Cells | 4.78 | 0.04 |
| ATM Signaling | 4.73 | 0.04 |
| Growth Hormone Signaling | 4.72 | 0.04 |
| FLT3 Signaling in Hematopoietic Progenitor Cells | 4.65 | 0.04 |
| Leptin Signaling in Obesity | 4.65 | 0.04 |
| VEGF Family Ligand-Receptor Interactions | 4.61 | 0.03 |
| Prostate Cancer Signaling | 4.52 | 0.03 |
| Xenobiotic Metabolism Signaling | 4.52 | 0.01 |
| Melanocyte Development and Pigmentation Signaling | 4.51 | 0.03 |
| Sumoylation Pathway | 4.5 | 0.03 |
| PAK Signaling | 4.43 | 0.03 |
| SAPK/JNK Signaling | 4.39 | 0.03 |
| Type I Diabetes Mellitus Signaling | 4.32 | 0.03 |
| Parkinson's Signaling | 4.31 | 0.13 |
| p53 Signaling | 4.31 | 0.03 |
| Paxillin Signaling | 4.28 | 0.03 |
| Pancreatic Adenocarcinoma Signaling | 4.23 | 0.03 |
| Fc Epsilon RI Signaling | 4.22 | 0.03 |
| Differential Regulation of Cytokine Production in Macrophages and T Helper Cells by IL-17A and IL-17F | 4.21 | 0.11 |
| LXR/RXR Activation | 4.2 | 0.02 |
| Role of Tissue Factor in Cancer | 4.19 | 0.02 |
| Atherosclerosis Signaling | 4.13 | 0.02 |
| Insulin Receptor Signaling | 4 | 0.02 |
| Differential Regulation of Cytokine Production in Intestinal Epithelial Cells by IL-17A and IL-17F | 3.99 | 0.09 |
| Ovarian Cancer Signaling | 3.97 | 0.02 |
| IL-22 Signaling | 3.95 | 0.08 |
| Role of JAK family kinases in IL-6-type Cytokine Signaling | 3.92 | 0.08 |
| Tec Kinase Signaling | 3.76 | 0.02 |
| Germ Cell-Sertoli Cell Junction Signaling | 3.74 | 0.02 |
| Sertoli Cell-Sertoli Cell Junction Signaling | 3.7 | 0.02 |
| NF-ÎºB Signaling | 3.68 | 0.02 |
| CREB Signaling in Neurons | 3.66 | 0.02 |
| Regulation of the Epithelial-Mesenchymal Transition Pathway | 3.62 | 0.02 |
| Role of NFAT in Cardiac Hypertrophy | 3.6 | 0.02 |
| Thrombin Signaling | 3.53 | 0.01 |
| Role of Hypercytokinemia/hyperchemokinemia in the Pathogenesis of Influenza | 3.44 | 0.05 |
| Integrin Signaling | 3.43 | 0.01 |
| LPS/IL-1 Mediated Inhibition of RXR Function | 3.42 | 0.01 |
| Cancer Drug Resistance By Drug Efflux | 3.33 | 0.04 |
| Docosahexaenoic Acid (DHA) Signaling | 3.28 | 0.04 |
| FcÎ³RIIB Signaling in B Lymphocytes | 3.26 | 0.04 |
| Melanoma Signaling | 3.23 | 0.04 |
| MSP-RON Signaling Pathway | 3.17 | 0.03 |
| G-Protein Coupled Receptor Signaling | 3.16 | 0.01 |
| Activation of IRF by Cytosolic Pattern Recognition Receptors | 3.12 | 0.03 |
| CNTF Signaling | 3.11 | 0.03 |
| ERK5 Signaling | 3.11 | 0.03 |
| PCP pathway | 3.11 | 0.03 |
| Endometrial Cancer Signaling | 3.1 | 0.03 |
| Pyridoxal 5'-phosphate Salvage Pathway | 3.08 | 0.03 |
| Hypoxia Signaling in the Cardiovascular System | 3.08 | 0.03 |
| Lymphotoxin Î² Receptor Signaling | 3.06 | 0.03 |
| Myc Mediated Apoptosis Signaling | 3.02 | 0.03 |
| Role of JAK1 and JAK3 in Î³c Cytokine Signaling | 3.01 | 0.03 |
| ErbB4 Signaling | 2.99 | 0.03 |
| STAT3 Pathway | 2.98 | 0.03 |
| GM-CSF Signaling | 2.98 | 0.03 |
| Antiproliferative Role of Somatostatin Receptor 2 | 2.96 | 0.03 |
| IL-15 Signaling | 2.95 | 0.03 |
| Role of PI3K/AKT Signaling in the Pathogenesis of Influenza | 2.95 | 0.03 |
| Non-Small Cell Lung Cancer Signaling | 2.94 | 0.03 |
| Macropinocytosis Signaling | 2.89 | 0.02 |
| PEDF Signaling | 2.86 | 0.02 |
| NF-ÎºB Activation by Viruses | 2.84 | 0.02 |
| OX40 Signaling Pathway | 2.81 | 0.02 |
| Apoptosis Signaling | 2.81 | 0.02 |
| Acute Myeloid Leukemia Signaling | 2.79 | 0.02 |
| Reelin Signaling in Neurons | 2.78 | 0.02 |
| Salvage Pathways of Pyrimidine Ribonucleotides | 2.76 | 0.02 |
| CDK5 Signaling | 2.72 | 0.02 |
| FAK Signaling | 2.72 | 0.02 |
| Antioxidant Action of Vitamin C | 2.69 | 0.02 |
| VEGF Signaling | 2.69 | 0.02 |
| Chronic Myeloid Leukemia Signaling | 2.68 | 0.02 |
| Mouse Embryonic Stem Cell Pluripotency | 2.66 | 0.02 |
| Gs Signaling | 2.64 | 0.02 |
| Glioma Signaling | 2.63 | 0.02 |
| Androgen Signaling | 2.62 | 0.02 |
| Telomerase Signaling | 2.62 | 0.02 |
| Nitric Oxide Signaling in the Cardiovascular System | 2.61 | 0.02 |
| p38 MAPK Signaling | 2.58 | 0.02 |
| PTEN Signaling | 2.56 | 0.02 |
| Synaptic Long Term Potentiation | 2.56 | 0.02 |
| fMLP Signaling in Neutrophils | 2.55 | 0.02 |
| Natural Killer Cell Signaling | 2.54 | 0.02 |
| Role of NANOG in Mammalian Embryonic Stem Cell Pluripotency | 2.54 | 0.02 |
| Sphingosine-1-phosphate Signaling | 2.53 | 0.02 |
| PI3K/AKT Signaling | 2.53 | 0.02 |
| FXR/RXR Activation | 2.51 | 0.02 |
| Estrogen Receptor Signaling | 2.5 | 0.02 |
| CCR3 Signaling in Eosinophils | 2.49 | 0.02 |
| p70S6K Signaling | 2.48 | 0.02 |
| Th2 Pathway | 2.37 | 0.01 |
| eNOS Signaling | 2.34 | 0.01 |
| Regulation of eIF4 and p70S6K Signaling | 2.33 | 0.01 |
| Glioblastoma Multiforme Signaling | 2.32 | 0.01 |
| Gq Signaling | 2.31 | 0.01 |
| Aldosterone Signaling in Epithelial Cells | 2.28 | 0.01 |
| Tight Junction Signaling | 2.27 | 0.01 |
| Gap Junction Signaling | 2.27 | 0.01 |
| Wnt/Î²-catenin Signaling | 2.26 | 0.01 |
| RhoGDI Signaling | 2.25 | 0.01 |
| Ephrin Receptor Signaling | 2.24 | 0.01 |
| Granulocyte Adhesion and Diapedesis | 2.23 | 0.01 |
| Calcium Signaling | 2.22 | 0.01 |
| Th1 and Th2 Activation Pathway | 2.19 | 0.01 |
| Agranulocyte Adhesion and Diapedesis | 2.17 | 0.01 |
| mTOR Signaling | 2.13 | 0.01 |
| Breast Cancer Regulation by Stathmin1 | 2.11 | 0.01 |
| EIF2 Signaling | 2.04 | 0.01 |
| cAMP-mediated signaling | 2.03 | 0.01 |
| Actin Cytoskeleton Signaling | 2.01 | 0.01 |
| Chondroitin Sulfate Degradation (Metazoa) | 1.98 | 0.06 |
| Phospholipase C Signaling | 1.98 | 0.01 |
| Dermatan Sulfate Degradation (Metazoa) | 1.95 | 0.06 |
| Endoplasmic Reticulum Stress Pathway | 1.86 | 0.05 |
| Inflammasome pathway | 1.86 | 0.05 |
| Estrogen-mediated S-phase Entry | 1.8 | 0.04 |
| Antiproliferative Role of TOB in T Cell Signaling | 1.77 | 0.04 |
| Circadian Rhythm Signaling | 1.66 | 0.03 |
| MIF-mediated Glucocorticoid Regulation | 1.66 | 0.03 |
| Oncostatin M Signaling | 1.65 | 0.03 |
| Coagulation System | 1.64 | 0.03 |
| Thyroid Cancer Signaling | 1.58 | 0.03 |
| Neuroprotective Role of THOP1 in Alzheimer's Disease | 1.58 | 0.03 |
| Protein Kinase A Signaling | 1.57 | 0.01 |
| Role of p14/p19ARF in Tumor Suppression | 1.55 | 0.02 |
| IL-9 Signaling | 1.53 | 0.02 |
| Graft-versus-Host Disease Signaling | 1.5 | 0.02 |
| Amyloid Processing | 1.48 | 0.02 |
| Semaphorin Signaling in Neurons | 1.46 | 0.02 |
| Axonal Guidance Signaling | 1.46 | 0 |
| Role of Cytokines in Mediating Communication between Immune Cells | 1.45 | 0.02 |
| Unfolded protein response | 1.45 | 0.02 |
| Regulation of Cellular Mechanics by Calpain Protease | 1.43 | 0.02 |
| Wnt/Ca+ pathway | 1.43 | 0.02 |
| Induction of Apoptosis by HIV1 | 1.41 | 0.02 |
| Ephrin A Signaling | 1.41 | 0.02 |
| Remodeling of Epithelial Adherens Junctions | 1.36 | 0.01 |
| Melatonin Signaling | 1.34 | 0.01 |
| Ephrin B Signaling | 1.32 | 0.01 |
| Angiopoietin Signaling | 1.3 | 0.01 |
| Small Cell Lung Cancer Signaling | 1.27 | 0.01 |
| HIPPO signaling | 1.26 | 0.01 |
| -Adrenergic Signaling | 1.25 | 0.01 |
| Bladder Cancer Signaling | 1.25 | 0.01 |
| Neuregulin Signaling | 1.25 | 0.01 |
| G Beta Gamma Signaling | 1.25 | 0.01 |
| HER-2 Signaling in Breast Cancer | 1.25 | 0.01 |
| Communication between Innate and Adaptive Immune Cells | 1.24 | 0.01 |
| IL-4 Signaling | 1.24 | 0.01 |
| Altered T Cell and B Cell Signaling in Rheumatoid Arthritis | 1.24 | 0.01 |
| Death Receptor Signaling | 1.23 | 0.01 |
| FcÎ³ Receptor-mediated Phagocytosis in Macrophages and Monocytes | 1.22 | 0.01 |
| TR/RXR Activation | 1.2 | 0.01 |
| CTLA4 Signaling in Cytotoxic T Lymphocytes | 1.2 | 0.01 |
| Virus Entry via Endocytic Pathways | 1.18 | 0.01 |
| Amyotrophic Lateral Sclerosis Signaling | 1.15 | 0.01 |
| Gi Signaling | 1.12 | 0.01 |
| iCOS-iCOSL Signaling in T Helper Cells | 1.11 | 0.01 |
| Phagosome Formation | 1.11 | 0.01 |
| Adipogenesis pathway | 1.07 | 0.01 |
| Th1 Pathway | 1.07 | 0.01 |
| Hereditary Breast Cancer Signaling | 1.05 | 0.01 |
| Human Embryonic Stem Cell Pluripotency | 1.04 | 0.01 |
| Synaptic Long Term Depression | 1.03 | 0.01 |
| Epithelial Adherens Junction Signaling | 1.03 | 0.01 |
| Dopamine-DARPP32 Feedback in cAMP Signaling | 0.99 | 0.01 |
| Mitochondrial Dysfunction | 0.97 | 0.01 |
| 3-phosphoinositide Biosynthesis | 0.92 | 0.01 |
| Clathrin-mediated Endocytosis Signaling | 0.91 | 0.01 |
| Superpathway of Inositol Phosphate Compounds | 0.85 | 0 |
